# Stage I–IV Colorectal Cancer Prognosis Can Be Predicted by Type and Number of Intratumoral Macrophages and CLEVER-1^+^ Vessel Density

**DOI:** 10.3390/cancers13235988

**Published:** 2021-11-28

**Authors:** Annika Ålgars, Lotta Kemppinen, Ruth Fair-Mäkelä, Harri Mustonen, Caj Haglund, Sirpa Jalkanen

**Affiliations:** 1 Department of Oncology, Turku University Hospital, 20521 Turku, Finland; 2 MediCity Research Laboratory and InFLAMES Flagship, Department of Microbiology and Immunology, University of Turku, 20520 Turku, Finland; riloke@utu.fi (L.K.); sirjal@utu.fi (S.J.); 3 Institute of Biomedicine, University of Turku, 20520 Turku, Finland; rufair@utu.fi; 4 Department of Surgery, University of Helsinki and Helsinki University Hospital, Haartmaninkatu 4, 00029 Helsinki, Finland; harri.mustonen@helsinki.fi (H.M.); caj.haglund@helsinki.fi (C.H.); 5 Translational Cancer Medicine Research Program, Faculty of Medicine, University of Helsinki, Haartmaninkatu 8, 00014 Helsinki, Finland

**Keywords:** colorectal cancer, macrophage, lymphatic vessel, CD68, CLEVER-1, survival

## Abstract

**Simple Summary:**

Tumor-associated macrophages can either promote or prevent cancer growth depending on factors such as macrophage polarization status, tumor type, and disease stage. Macrophages and vessels interact with each other, and the number of lymphatic vessels also affects cancer survival. CLEVER-1 is a protein expressed both on immunosuppressive M2 macrophages and lymphatic vessels. The aim of this study was to validate our previous results regarding the prognostic role of CLEVER-1^+^ macrophages, CD68^+^ macrophages, and CLEVER-1^+^ lymphatic vessels in stage I–IV colorectal cancer. The results indicate that the prognostic role of tumor-associated macrophages and lymphatic vessels changes during disease progression. The findings resemble our earlier results, but are not completely equal, which may be due to the different types of tumor samples used in the two studies (whole section vs. tissue microarray).

**Abstract:**

Macrophages, which are key players in the tumor microenvironment and affect the prognosis of many cancers, interact with lymphatic vessels in tumor tissue. However, the prognostic role of tumor-associated macrophages (TAM) and lymphatic vessels in human colorectal cancer (CRC) remains controversial. We investigated the prognostic role of CD68^+^ and CLEVER-1^+^ (common lymphatic endothelial and vascular endothelial receptor 1) TAMs in addition to CLEVER-1^+^ lymphatic vessels in 498 stage I–IV CRC patients. The molecular markers were detected by immunohistochemical (IHC) analysis. The results showed that, in early stage I CRC and in young patients (age below median, ≤67.4 years), a high number of CD68^+^ and CLEVER-1^+^ TAMs was associated with longer disease-specific survival (DSS). In early stage I CRC, high intratumoral CLEVER-1^+^ lymphatic vessel density (LVD) predicted a favorable prognosis, whereas the opposite pattern was observed in stage II CRC. The highest density of CLEVER-1^+^ lymphatic vessels was found in metastatic disease. The combination of intratumoral CLEVER-1^+^ lymphatic vessel^high^ + CD68^+^ TAM^low^ was associated with poor DSS in stage I–IV rectal cancer. The present results indicate that the prognostic significance of intratumoral macrophages and CLEVER-1^+^ lymphatic vessels differs according to disease stage, reflecting the dynamic changes occurring in the tumor microenvironment during disease progression.

## 1. Introduction

**Figure 1:**
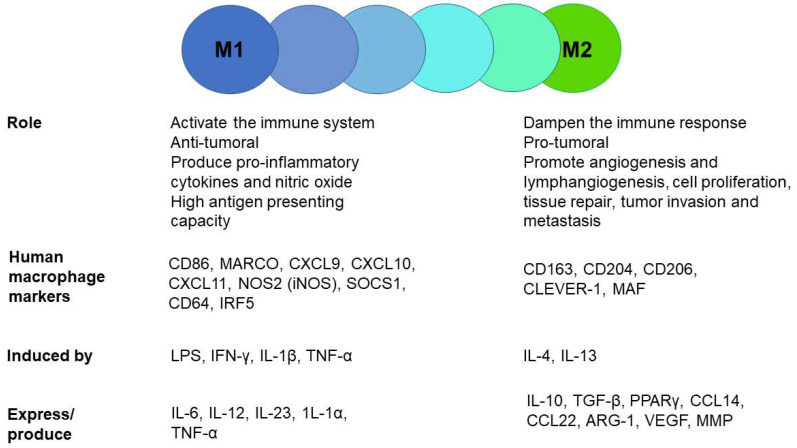
The role and characteristics of M1 and M2 macrophages representing the two extremes of the macrophage polarization spectrum.

CRC, one of the most common cancers globally, has a high mortality rate and increasing incidence [1]. A connection between inflammation and CRC exists [2,3,4]. Inflammatory bowel diseases increase the risk of malignant transformation in the gut, and nonsteroidal anti-inflammatory drugs such as acetylsalicylic acid have antitumor effects [5,6,7]. TAMs, among other inflammatory cells, infiltrate tumor tissue and polarize into different phenotypes depending on the tumor microenvironment [8,9]. Cytotoxic, pro-inflammatory M1 macrophages prevent tumorigenesis, in contrast to M2 macrophages, which are anti-inflammatory, and therefore pro-tumoral. These two types of macrophages represent the two extremes of the macrophage polarization spectrum. TAMs are plastic cells that can switch phenotype or express both M1 and M2 markers [10,11,12,13,14,15]. In the early stage of disease, TAMs are mainly M1 type and polarize to M2 type during disease progression. TAMs promote tumor cell proliferation, invasion, and migration; enhance angiogenesis and lymphangiogenesis; suppress T cells; and affect the gut microbiota [16]. Markers used for M2 macrophage detection include CD163 (macrophage scavenger receptor), CD204 (macrophage scavenger receptor 1), CD206 (macrophage mannose receptor), and CLEVER-1 [15,17,18,19]. The role and characteristics of M1 and M2 macrophages have been presented in Figure 1.

CLEVER-1, also called stabilin-1 or FEEL-1 is a multifunctional transmembrane protein expressed on the surface of a subset of immunosuppressive M2 macrophages and monocytes, afferent and efferent lymphatic endothelium, sinusoidal endothelial cells in the liver and spleen, high endothelial venules (HEVs) and HEV-like vessels during inflammation and cancer [20,21,22]. We previously investigated the co-expression of CLEVER-1 and another marker for M2 macrophages, macrophage mannose receptor (MR). We showed that MR^+^ macrophages appear earlier in tumors than CLEVER-1^+^ macrophages which appear later during cancer growth and only in a subset (about 50%) of MR^+^ macrophages [18]. In addition, we previously reported that 95% of the MR^+^ macrophages co-express CLEVER-1 in human CRC tissue [19]. CLEVER-1 is involved in multiple functions, including cell trafficking within lymphatic vessels, scavenging and tissue remodeling [23].

The prognostic significance of TAMs has been studied in several human cancer types, and according to previous reports, a high density of TAMs predicts poor patient survival in many cancers [24,25,26,27,28]. In CRC, contradictory results regarding the prognostic role of TAMs have been reported [16,19,29,30,31,32,33,34,35,36,37,38], Table 1.

In vivo studies show that CLEVER-1 knockout mice have a lower number of immunosuppressive leukocytes in their tumors and smaller primary tumors and metastases, than wild-type mice. In addition, anti-CLEVER-1 treated mice had smaller primary tumors and metastases than the controls [18]. The prognostic/predictive significance of CLEVER-1 has been studied retrospectively in human bladder cancer, head and neck cancer, and CRC. In bladder cancer, the combination of a high number of CD68^+^ and CLEVER-1^+^ or MAC387^+^ and CLEVER-1^+^ macrophages predicts a shorter survival [39]. In a series of 54 head and neck cancer patients, elevated expression of stabilin-1 (CLEVER-1) was associated with shorter recurrence-free survival [40]. However, the precise role of CLEVER-1^+^ M2 macrophages in tumorigenesis remains unknown.

The prognostic significance of lymphatic vessels in CRC depends on the location of the vessels within the tumor. Our group and others showed that a high number of intratumoral podoplanin^+^ lymphatic vessels have tumorigenic effects in CRC [19,41]. This could be explained by the fact that the vessels serve as a route for metastasis. Peritumoral CLEVER-1^+^ and podoplanin^+^ lymphatic vessels, on the other hand, seem to have anti-tumor effects and associate with improved prognosis [19]. However, published results regarding the prognostic role of lymphatic vessels in CRC remain inconclusive [42,43].

In a previous study, we investigated the prognostic significance of CD68^+^ and CLEVER-1^+^ macrophages and lymphatic vessels in 159 stage II–IV CRC patients. We reported that both the subtype and location of macrophages as well as the density and location of lymphatic vessels within the tumor microenvironment, affect the outcome of CRC. The prognostic role of TAMs change according to tumor stage [19].

The outcome of patients with CRC has improved, which is at least partly due to the availability of new prognostic and predictive markers. These have improved the tailoring of treatment strategies, including the accurate selection of patients for specific treatments. The prognostic factors vary according to the stage of the disease. Established prognostic factors in early stage disease include preoperative intestinal obstruction or perforation, number of lymph nodes sampled, pT stage, microsatellite instability (MSI) status, lymphovascular or perineural invasion, tumor differentiation grade, and carcinoembryonic antigen levels [44]. Stage II–III MSI-high (MSI-H) tumors are immunologically active and these patients have an excellent prognosis [45,46]. *BRAF* mutational status and the location of the primary tumor are factors associated with disease outcome in advanced stage IV disease [47,48]. Immune checkpoint inhibitors have become available for the treatment of different cancer types [45], including metastatic MSI-H stage IV CRC disease, in which anti-PD-1 and anti-CTLA-4 therapies have shown efficacy in phase II and III studies [49,50,51,52,53,54]. Other drugs that target the immune system are being investigated; bexmarilimab, an anti-CLEVER-1 antibody, is being tested in the MATINS phase I/II trial which has shown encouraging preliminary results. The trial population comprises patients with advanced or metastatic solid tumors including CRC patients [55,56,57,58].

The aim of this study was to extend our previous studies regarding the prognostic significance of intratumoral CD68^+^ TAMs, CLEVER-1^+^ TAMs, and CLEVER-1^+^ LVD in a larger stage II–IV CRC patient population. In addition, we evaluated the prognostic significance of the above-mentioned factors in a new subgroup of patients with stage I CRC. The value of tissue microarray (TMA) tumor samples in a study setting such as the present one was also evaluated. The association of TAMs and CLEVER-1^+^ vessel number with pre-defined clinicopathological variables was investigated.

## 2. Materials and Methods

### 2.1. Patients

**Figure 2:**
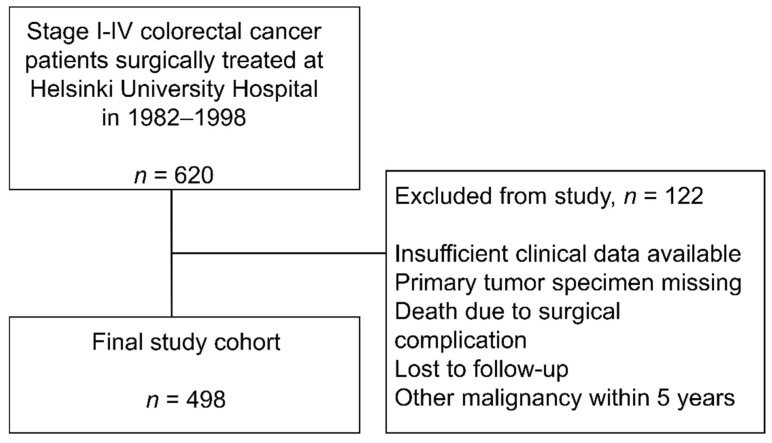
A flow diagram showing the inclusion and exclusion of the patients to this study.

A cohort of 620 patients diagnosed and surgically treated at the Helsinki University Hospital between 1982 and 1998 for stage I–IV CRC was used for this study. Clinical data and tumor specimens were available for 498/620 (80.3%) of the patients. Patient inclusion and exclusion to the study are shown in Figure 2.

The median age of the patients was 67.4 years (22.7–90.3 years). The majority of the patients had stage II CRC. Additional patient characteristics are presented in Table 2. The median follow-up time was 4.81 years, and 46% (229/498) of the patients died during the follow-up period. Survival data, including cause of death, were received from the Population Register Centre of Finland and Statistics Finland.

This retrospective study was conducted in accordance with the Declaration of Helsinki. The Ethics Committee at the University of Helsinki approved the study (226/E6/2006, extension 17 April 2013). The approval to conduct the retrospective study came from the National Supervisory Authority of Welfare and Health (Valvira Dnro 10041/06.01.03.01/2012) and is supported by the following Finnish laws: Act on the Medical Use of Human Organs, Tissues and Cells (No. 101/2001: §20 (1), §22 (2), and §23, and No. 594/2001: §12 and §13). Permission to use fresh frozen CRC tissue for investigation of lymphatic vessels in CRC was granted by the Research Ethics Board of the Hospital District of Southwest Finland (T175/2016). Written consent was obtained from the participants.

### 2.2. Tissue Samples

Tumor specimens were collected during routine surgery prior to the initiation of systemic therapy. The specimens were fixed with 10% formalin following the clinical standard procedures of the Department of Pathology. The specimens were embedded in paraffin and stored at room temperature for preservation. Tissue microarray blocks were constructed by punching three representative cores (1.0 mm) from each tumor using a semiautomatic tissue microarrayer (Tissue Arrayer 1, Beecher Instruments Inc., Silver Springs, MD, USA). Fresh frozen CRC tissue was collected during routine primary tumor surgery. The tumor samples were embedded in optimal cutting temperature (O.C.T) medium and snap frozen on dry ice. The samples were cut into 5 µm cryostat-sections and fixed with acetone.

### 2.3. Immunohistochemistry

Macrophages were identified by detecting CD68, a macrophage marker, using a mouse monoclonal IgG1 anti-CD68 pre-diluted ready-to-use antibody (Abcam, Cambridge, UK, ab845), and 3G6 (10 µg/mL) served as a negative control. 2–7 (rat IgG) antibody (10 µg/mL) was used to detect CLEVER-1 expressing M2 macrophages and lymphatic vessels. MEL-14 (10 µg/mL) against mouse L-selectin was used as a negative control.

Before staining, the tumor blocks were cut into 4 µm sections, deparaffinized in xylene and rehydrated with a graded alcohol series. A two-step IHC staining technique was used to detect CD68 and CLEVER-1 markers in the TMA samples. Vectastain Elite ABC mouse (Vector Laboratories, Inc., Burlingame, CA 94010, USA, PK-6102) and rat (Vector Laboratories, PK-6104) kits were used for anti-CD68 and CLEVER-1 stainings, respectively, according to the manufacturer’s instructions. For anti-CD68 and CLEVER-1 staining, antigen retrieval was performed with Proteinase K (Dako North America, Inc., Carpinteria, CA 93013, USA, S3020). Endogenous peroxidase was blocked using H_2_O_2_, and non-specific staining was prevented by incubating, samples in normal serum from Vectastain Elite kits (Vector Laboratories, Inc., Burlingame, CA 94010, USA). DAB chromogen (Dako North America, Inc., Carpinteria, CA 93013, USA, K3468) was used for detection. Mayer’s hematoxylin was used for counterstaining. The laboratory staff performed the analyses blinded to the clinical outcomes of the patients.

### 2.4. Immunofluorescence Staining and Evaluation of Stainings

Primary antibodies detecting lymphatic vessels (anti-Lyve1, ReliaTech, 38300 Wolfenbüttel, Germany, 102-PA50AG, 10 µg/mL) and CLEVER-1 expressing cells (2–7 antibody, 10 µg/mL) were incubated overnight at +4 °C. Primary antibodies were detected with fluorescently labeled Goat anti-rabbit IgG Alexa Fluor 546 (Invitrogen, Life Technologies Corporation, Eugene, OR 97402, USA, A11035) and Goat anti-rat IgG Alexa Fluor 488 (Invitrogen, A11006) secondary antibodies (5 µg/mL in PBS supplemented with 5% normal human serum), followed by staining of macrophages with Alexa Fluor 647 conjugated CD68 antibody (Santa Cruz Biotechnology Inc, Dallas, TX 75220, USA, sc-20060) all incubated for 1 h at room temperature. Sections were overlaid with ProLong Gold mounting medium containing DAPI (Invitrogen, Life Technologies Corporation, Eugene, OR 97402, USA, P36930). For control staining, samples were overlaid with rabbit IgG (Sigma-Aldrich, St Louis, MO 63103, USA, 15006, 10 µg/mL) and MEL-14 (rat IgG antibody detecting murine CD62L, negative staining in human, 10 µg/mL; kind gift from E. Butcher, Stanford University, Stanford, CA 94305, USA) followed by the same secondary antibodies as above and a directly conjugated Alexa Fluor 647 mouse IgG1 isotype control (Invitrogen, Life Technologies Corporation, Eugene, OR 97402, USA, BD 557783, 1:50).

Samples were examined with a LSM 880 confocal microscope (Carl Zeiss Microscopy GmbH, Jena, Germany) using the Plan-Apochromat ×20/0.8 objective and acquired with the Zen 2.3 SP1 FP2 software version 14.0.22.201 (Carl Zeiss Microscopy GmbH). Pinhole adjustments were identical for all channels. Image analysis was performed using the open software Fiji [59]. Background subtraction and linear brightness adjustments were applied equally to all images.

### 2.5. Evaluation of CD68 and CLEVER-1 Expression

Stained TMA specimens were evaluated semiquantitatively using a light microscope (Olympus BX60, Olympus Corporation, Tokyo, Japan; ×200 and ×400 magnifications). Samples were classified into four categories according to the quantity of CD68 and CLEVER-1 antigen-expressing macrophages and vessels/TMA spot. The scoring was performed independently by two observers (L.K. and A.Å.) who were blinded to the clinical information of the patients.

For the macrophage marker, CD68, the categories used were 0 (none, 0 macrophages/TMA spot), 1 (low, 1–29 macrophages/TMA spot), 2 (moderate, 30–99 macrophages/TMA spot), and 3 (high, >100 macrophages/TMA spot). The classification for CLEVER-1^+^ macrophages was 0 (none, 0 macrophages/TMA spot), 1 (low, 1–9 macrophages/TMA spot), 2 (moderate, 10–30 macrophages/TMA spot), and 3 (high, >30 macrophages/TMA spot). The semiquantitative grading score for lymphatic vessels was: none (−), a few (+), moderate (++), and abundant (+++). If the grading of the two observers was discordant, samples were reviewed by both observers together until an agreement was reached. The highest and mean macrophage and vessel scores detected from the TMA spots were used for statistical analyses.

### 2.6. Statistical Analysis

SPSS statistics v 24 (IBM Corporation, Armonk, NY, USA) was used for statistical analyses. The relationship of the different subsets of macrophages and CLEVER-1^+^ vessels with clinicopathological variables and disease outcome was evaluated. Frequency table data were analyzed with the χ2-test or Fisher’s Exact Test. Univariate survival analysis was performed using the Kaplan-Meier method and log-rank test. Death from CRC was considered the endpoint in the analysis of DSS. Multivariate survival analysis was performed using the Cox proportional hazards model. The assumption of constant hazard ratios over time was tested by including time dependent covariates for each testable variable. Stratified multivariate analysis was performed for differentiation grade (3–4 vs. 1–2) and stage (III–IV vs. I–II), if needed.

The prognostic effect of the macrophages and vessels was pre-planned for investigation in the following subgroups: women vs. men, colon vs. rectum, right vs. left-sided primary tumor location, differentiation grade of the tumors, and different stage groups. *p*-values < 0.05 were considered statistically significant. All statistical tests were two-sided.

## 3. Results

### 3.1. A High Number of CD68^+^ Macrophages Is Commonly Detected in CRC Tumor Samples

**Figure 3:**
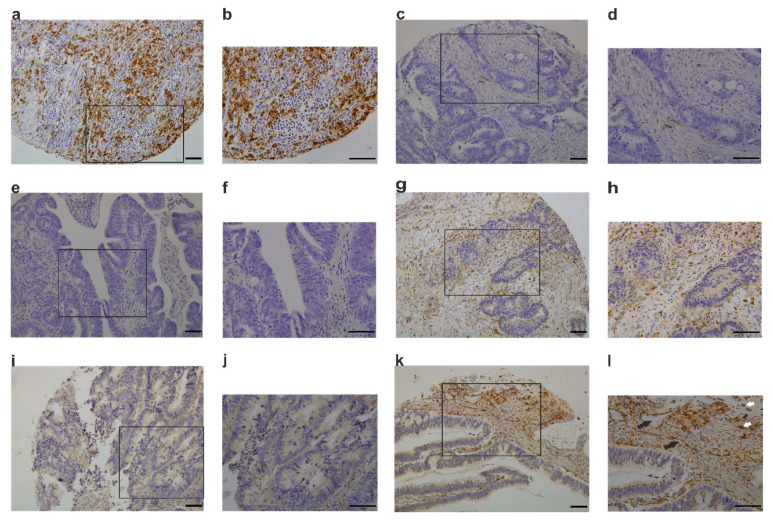
Immunohistochemical stainings of CLEVER-1^+^/CD68^+^ macrophages and CLEVER-1^+^ lymphatic vessels in intratumoral stage I–IV colorectal cancer (CRC) samples. (**a**,**b**) High CD68^+^ macrophage number. (**c**,**d**) CRC sample with a low CD68^+^ macrophage number. (**e**,**f**) CRC sample lacking CD68^+^ macrophages. (**g**,**h**) High CLEVER-1^+^ macrophage number. (**i**,**j**) Sample lacking CLEVER-1^+^ macrophages. (**k**,**l**) CRC sample with high amount of both CLEVER-1^+^ macrophages and vessels. Black arrows indicate vessels, white arrows indicate macrophages. Magnification ×10 in (**a**,**c**,**e**,**g**,**i**,**k**) magnification ×20 in insets (**b**,**d**,**f**,**h**,**j**,**l**). Scale bars 100 µm in all figures.

**Figure 4:**
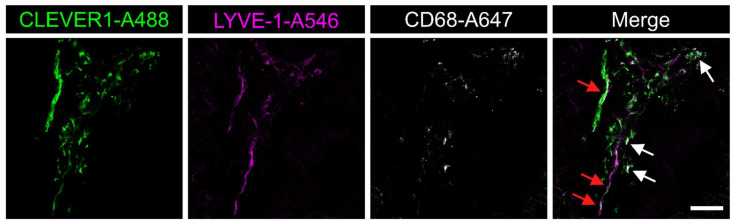
Immunofluorescence staining of CLEVER-1^+^/CD68^+^ macrophages and CLEVER-1^+^ lymphatic vessels in a fresh frozen colorectal cancer sample. White arrows show CLEVER-1^+^/CD68^+^ macrophages, whereas red arrows mark LYVE-1^+^ lymphatic vessels positive for CLEVER-1. Scale bar 50 µm.

The number of CLEVER-1^+^ M2 macrophages was lower than that of CD68^+^ macrophages in CRC samples. The majority of samples had a high number (score category 3) of CD68^+^ or CLEVER-1^+^ macrophages. Few samples were devoid of CD68^+^ macrophages, CLEVER-1^+^ macrophages, or CLEVER-1^+^ lymphatic vessels. Of those, the lack of CD68^+^ macrophages was more common than the lack of CLEVER-1^+^ macrophages (6.8% vs. 1.0%). Although the difference was not statistically significant, this result indicates that CD68-negative and CLEVER-1-positive macrophages exist. The macrophage and vessel scores are presented in Table 3. Examples of IHC stainings are shown in Figure 3. CLEVER-1^+^ vessels were shown to co-express LYVE-1 and CLEVER-1^+^ macrophages CD68 (Figure 4).

### 3.2. Intratumoral CD68^+^ or CLEVER-1^+^ Macrophage Number Is Not Associated with DSS

In the analysis of the entire patient cohort, the factors associated with poor DSS were advanced stage of disease [hazard ratio (HR) 2.0, 95% confidence interval (CI): 1.02–3.8, *p* = 0.044; HR: 5.9, 95% CI: 3.1–11.1, *p* < 0.001; and HR: 22.9, 95% CI: 12.1–43.1, *p* < 0.001; for stages II, III, and IV vs. I, respectively], poor tumor differentiation grade (HR: 1.6, 95% CI: 1.2–2.1, *p* = 0.001; Grade 3–4 vs. 1–2) and age above median (HR: 1.5, 95% CI: 1.1–1.9, *p* = 0.004).

Stage I CRC patients with high density of CD68^+^ macrophage infiltration in primary tumors had a mean DSS of 25.1 (95% CI: 23.0–27.3) years, and patients with <100 CD68^+^ macrophages detected/TMA spot had a DSS of 23.0 (95% CI: 18.4–27.5) years, although the difference was not significant (log-rank *p* = 0.12). In stage IV metastatic disease a high CLEVER-1^+^ macrophage number was associated with numerically shorter DSS, although this finding did not reach statistical significance either [high 1.4 (95% CI: 0.9–2.2) years vs. others 4.6 (95% CI: 1.4–7.4) years, log-rank *p* = 0.055].

### 3.3. CLEVER-1^+^ Lymphatic Vessel Number Is Increased in Metastatic CRC

A positive correlation was observed between CLEVER-1^+^ LVD and CD68^+^ macrophage number (Spearman rho 0.172, SE 0.047, *p* < 0.0001). In addition, CLEVER-1^+^ and CD68^+^ macrophage number correlated negatively with each other (Spearman rho −0.115, SE 0.047, *p* = 0.018).

A high CLEVER-1^+^ lymphatic vessel number was more common in stage IV metastatic disease (57/99, 57.6%) than in stage I-III disease (158/351, 45.0%; Fischer´s exact test, *p* = 0.03). No association was observed between CLEVER-1^+^ lymphatic vessel number and tumor differentiation grade, gender, tumor location, or age at diagnosis.

### 3.4. The Prognostic Significance of CLEVER-1^+^ Lymphatic Vessel Density Differs According to the Stage of Disease

**Figure 5:**
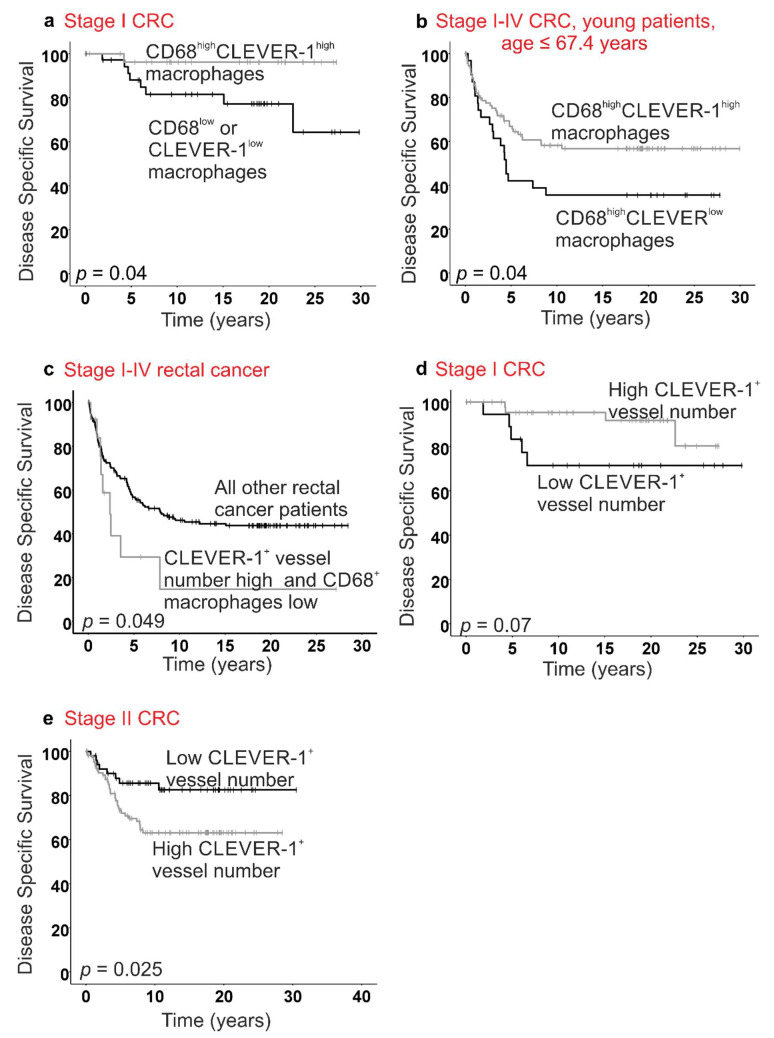
Kaplan-Meier survival curves of disease-specific survival (DSS) in colorectal cancer (CRC) patients. (**a**) In stage I CRC, high number of both CD68^+^ and CLEVER-1^+^ macrophages was associated with improved DSS. (**b**) In young patients (age below median, ≤67.4 years) with a high CD68^+^ macrophage density, a low CLEVER-1^+^ macrophage number was associated with poor outcomes. (**c**) A high density of CLEVER-1^+^ lymphatic vessels together with a low number of CD68^+^ macrophages was associated with poor survival in rectal cancer. (**d**) In stage I CRC a high number of CLEVER-1^+^ lymphatic vessels was a sign of good prognosis, which was opposite to stage II CRC (**e**), where a high CLEVER-1^+^ LVD was associated with poor outcome.

A high density of intratumoral CLEVER-1^+^ lymphatic vessels was associated with poor DSS in stage II CRC (log-rank, *p* = 0.025, Figure 5e) in contrast to stage I disease, in which patients with a high number of CLEVER-1^+^ lymphatics tended to have longer survival, although the difference was not significant (log-rank, *p* = 0.07, Figure 5d). In stage I disease, CLEVER-1^+^ lymphatic vessel number was a significant prognostic factor in multivariate analysis adjusted for age, sex, location, type (adeno/mucinous) and differentiation status (HR: 0.23, 95% CI: 0.06–0.94, *p* = 0.041), but not in stage II disease (HR: 2.0, 95% CI: 0.92–4.4, *p* = 0.08).

### 3.5. A Combined Score Is Associated with Survival

In stage I disease, the combination of high numbers of CD68^+^ and CLEVER-1^+^ macrophages was associated with improved survival (log-rank *p* = 0.04, Figure 5a). A similar finding was obtained when analyzing the subgroup of young (age below median) patients (log-rank *p* = 0.04, Figure 5b).

In stage I-IV rectal cancer patients, a high number of CLEVER-1^+^ lymphatic vessels in combination with a low number of CD68^+^ macrophages was associated with poor survival (log-rank *p* = 0.049; Figure 5c). The combined score remained statistically significant in multivariate survival analysis adjusted for age, sex, type (adeno/mucinous), differentiation status, and stage (Cox *p* = 0.015; HR: 2.4; 95% CI: 1.19–4.9).

## 4. Discussion

In the present study, we showed that the presence of a high number of both CD68^+^ and CLEVER-1^+^ macrophages intratumorally was associated with favorable disease outcome in early stage I CRC and in the subgroup of younger patients (age below median). The association between CLEVER-1^+^ lymphatic vessel number and survival differed according to the stage of the disease. A high CLEVER-1^+^ intratumoral lymphatic vessel number was associated with poor disease outcome in stage II CRC, in contrast to patients with early stage I disease.

Previous studies reported an association between a high TAM, especially CD68^+^ macrophage, number and improved survival in CRC [19,30,31,32,33,34]. The present results, regarding CD68^+^ TAMs in stage I disease, were consistent with previous data, although they failed to reach statistical significance (*p* = 0.12). Opposite results have also been published [16]. Studies investigating TAMs in CRC are heterogeneous regarding macrophage markers and cut-offs used, the distribution of macrophages within the tumor tissue (e.g., intra- vs. peritumoral), and stage of disease amongst other factors, which may explain the controversy in this field.

The findings of this study indicate that in early-stage CRC with an excellent prognosis, the role of intratumoral CLEVER-1^+^ TAMs differs from the pro-tumoral role of M2 TAMs. Similar results were previously reported by other groups. Edin et al. [33], showed that a high number of CD163^+^ M2 TAMs at the tumor front was associated with improved survival in stage I–IV CRC [33]. A study by Koelzer et al. [34], found an association between a high CD163^+^ TAM number and factors associated with favorable disease outcome, such as a lower incidence of lymph node metastasis and less advanced T-stage; however, a statistically significant survival benefit was not demonstrated for these patients, although a difference in survival time was observed [34].

In stage IV disease, a high intratumoral CLEVER-1^+^ macrophage number was associated with a numerically shorter DSS, although the finding was only borderline significant. This result is in line with our previous findings, which showed that a high intratumoral CLEVER-1^+^ macrophage number had a negative impact on DSS in metastatic stage IV disease. In our previous study, high peritumoral CLEVER-1^+^ macrophage density was associated with improved DSS in non-metastatic stage II and III CRC [19]. We investigated the prognostic role of intratumoral CD68^+^ and CLEVER-1^+^ macrophages in the subgroup of stage II–III CRC in this study as well, but no statistically significant findings were obtained. The present study differs from our previous study by including stage I CRC patients and by including only intratumoral TMA samples, which excluded investigation of the peritumoral area.

A study that included 209 stage I–III CRC patients showed that a high number of CD163^+^ TAMs had a negative effect on survival. Similar to the present study, the analysis was based on TMA samples, and different areas of CRC tissue were not separately analysed; in addition, subgroup analysis according to stage was not performed [36]. Inagaki et al. [37], studied CD68^+^CD163^−^ (M1) and CD68^+^CD163^+^ (M2) macrophages in four different CRC tumor regions (invasive front, tumor center, lateral periphery of the tumor, and healthy mucosa adjacent to the tumor) and showed a dynamic increase in M2 TAM number and M2/M1 ratio at the invasive front during cancer progression [37]. A high CD163^+^/CD68^+^ TAM ratio at the tumor front was associated with worse survival in a study investigating Stage I–III CRC (*n* = 81), whereas no such association was observed when a high CD163^+^/CD68^+^ TAM ratio was found intratumorally [38]. The findings of previous studies regarding the prognostic role of TAMs in CRC have been summarized in Table 1.

The distribution of TAMs within the tumor microenvironment clearly affects the prognostic role of TAMs and should be considered when designing future studies. In addition, the use of several macrophage markers and measuring their ratio is a promising approach [19,35,37,38].

We previously reported that the prognostic effect of lymphatic vessel number varies according to the stage of CRC as well as the localization of lymphatic vessels within the tumor microenvironment [19]. The results presented here suggest that the prognostic role of CLEVER-1^+^ vessels varies in a stage-dependent manner as well. A study by Barresi et al. [60] focusing on stage I CRC showed that a high peritumoral LVD is associated with poor disease-free survival. However, no such association was found when investigating intratumoral LVD. The present findings regarding intratumoral CLEVER-1^+^ lymphatic vessels in stage I disease are opposite to the results of Barresi et al. [60], who used D2-40 (podoplanin) as a lymphatic marker [60]. To the best of our knowledge, the prognostic role of CLEVER-1^+^ LVD has not been previously investigated in stage I CRC. In this study, a positive correlation was observed between CD68^+^ macrophage number and CLEVER-1^+^ vessel density. This finding was expected because of the known interaction between TAMs and vascular cells. TAMs promote angiogenesis and lymphangiogenesis in tumor tissue by several mechanisms, such as the secretion of pro-angiogenic factors including vascular endothelial growth factors [15,61].

In this study, a low CD68^+^ macrophage number in combination with a high CLEVER-1^+^ LVD was associated with poor survival in rectal cancer. Lackner et al. [30], reported that a high microvessel number in combination with low CD68^+^ macrophage density at the invasive tumor front was a sign of poor prognosis in stage II CRC, which is in line with the present findings [30]. In vivo studies show that the presence of CLEVER-1 in the vessel endothelium enables tumor cell spread, which may explain the present results [18].

A gene expression-based classification of CRC by an international consortium divides CRC patients into four subgroups, namely, CMS1–CMS4. Tumors belonging to the CMS1 group are immunologically active and show anti-tumoral/anti-inflammatory effects, and they are often located on the right side of the gut. Tumors belonging to the mesenchymal CMS4 subgroup are angiogenic and infiltrated by inflammatory cells which possess pro-tumoral effects and are associated with poor survival. Left-sided CRC tumors commonly belong to the CMS2 subgroup and are regarded as immunologically “cold” tumors [62,63,64]. Although CMS subgroup information of the patients was lacking in this study, a correlation between tumor location (left vs. right-sided) and macrophage number, macrophages subtype, or vessel number was expected, however no such association was observed. MSI-H CRC tumors are often right-sided, immunologically active, belong to the CMS1 subgroup, and have a favorable prognosis [45,46,62]. A high TAM number was associated with improved overall survival in microsatellite stable (MSS), but not MSI-H CRC, in a meta-analysis by Li et al. [32]. By contrast, Väyrynen et al. [35] analyzed a patient cohort of 931 CRC patients and showed that MSI-H CRC tumors have a higher number of TAMs than MSS tumors and that a high total number of stromal macrophages or M1 macrophages is associated with improved survival in MSI-H CRC [35]. MSS/MSI status, as well as Lynch syndrome testing results, are missing from the present study because these analyses were not routinely performed during the inclusion period, in contrast to current routine practice. Therefore, the prognostic impact of CLEVER-1^+^ TAMs, CD68^+^ TAMs, or CLEVER^+^ lymphatic vessels could not be evaluated in these subgroups in this study. Our results showing a favorable prognosis in younger patients with a high number of intratumoral CD68^+^CLEVER-1^+^ TAMs may be linked with hereditary Lynch syndrome, which is characterized by mutations in the MMR genes leading to MSI-H tumors. Lynch syndrome is diagnosed in 2–4% of CRC patients. CRC tumors associated with Lynch syndrome are often right-sided and diagnosed at an earlier age than sporadic CRC.

This study was designed to investigate the prognostic role of CD68^+^, CLEVER^+^ macrophages, and CLEVER^+^ lymphatic vessels in CRC; however, the predictive role of these factors could not be examined because of the lack of detailed cancer treatment data for our patient cohort. Advances in the treatment of CRC and the development of new drugs, especially those for the treatment of patients with stage IV disease, occurred after the inclusion period of this study. Therefore, the predictive role of these markers should be evaluated in a newer patient cohort in the future.

Approved immunotherapies for CRC are indicated only for metastatic stage IV disease [53,54]. Preliminary results from the MATINS study show that treatment with FP-1305 (bexmarilimab) antibody targeting CLEVER-1 changes the tumor microenvironment from immunosuppressive to antitumoral by, for example, switching the macrophage polarization from M2 to M1 type; increasing the level of interferon gamma, circulating CD8^+^ T cells, and natural killer cells; and decreasing the level of regulatory T-cells. The treatment shows promising antitumor activity with an acceptable toxicity profile [55,56,57,58]. The present results regarding the prognostic role of CLEVER-1 in CRC indicate that anti-CLEVER-1 therapy may show the greatest benefits in CRC patients in the metastatic stage of the disease. In the MATINS trial the highest levels of IFNγ following anti-CLEVER-1 therapy was observed in a patient with metastatic MSS CRC. This patient had a partial response to the treatment [55,58]. The predictive role of CLEVER-1 during immune check point inhibitor therapy has not been prospectively studied. The Cancer Genome Atlas pan-cancer cohort data indicate that high expression of CLEVER-1 is associated with a poor response to immunotherapy and shorter survival [23]. Other treatment approaches targeting TAMs include reprogramming M2 macrophages to M1 (e.g., using low doses of radiation, targeting toll-like receptors, or use of zoledronic acid) or depleting existing M2 macrophages (e.g., by targeting the CSF-1/CSF-1R pathway), which cause resistance to cancer therapies, preventing TAM recruitment to tumor tissue (e.g., using anti-CCL2 antibodies or CCR2 inhibitors), and preventing TAM-mediated angiogenesis (via anti-VEGF antibody) [15]. Combination therapies targeting both angiogenesis and TAMs are also being investigated (e.g., Ang-2 and VEGF inhibitors; dual Ang-2/VEGF inhibitors and PD-L1 or CD40 immunotherapy) [61].

The large sample size, as well as the inclusion of both early stage and metastatic CRC patients, are among the strengths of this study. The retrospective study design might, on the other hand, have an impact on the results, which should be regarded as hypothesis-generating rather than practice-changing. Taken together with previous data, the present results indicate that macrophage number, subtype, distribution, as well as lymphatic vessel number and location within the tumor microenvironment, impact CRC prognosis. The underlying mechanisms are complex, and the discrepant results published on this subject further complicate the picture and prevent us from drawing firm conclusions from our results. The present results are consistent with our previously published results on this subject, although they are not completely equal. These discrepancies may be due to the molecularly heterogeneous nature of CRC tumor tissue [65], and therefore TMA specimens might not be the optimal tumor tissue type for this kind of CRC biomarker studies. The use of TMA samples requires thorough preselection of the tissue area of interest for each specific marker to be analyzed.

## 5. Conclusions

In conclusion, this study shows that the prognostic role of M1 and M2 macrophages and CLEVER-1^+^ lymphatic vessels in CRC varies according to disease stage. The present results underscore the need for standardized quantification methods and indicate that TMA tumor samples, at least without careful punching area selection, might not be the optimal method for this type of study.

## Figures and Tables

**Table 1:** The prognostic role of tumor associated macrophages in colorectal cancer according to previous studies.

Study [Reference]	Number of Patients	Stage of Disease	Macrophage Markers Used	Findings
Ålgars et al. [19]	159	II, III, IV	CLEVER-1 CD68	High CD68^+^ TAM number PT → improved DSS High CLEVER-1^+^ TAM number PT → improved DSS in stage III CRC High CLEVER-1^+^ TAM number IT → shorter DSS in stage IV CRC High CLEVER-1^+^ TAM number PT → improved DSS in stage III CRC, and decreased survival in stage IV CRC
Cavnar et al. [29]	158	IV	CD68	High CD68^+^ TAM number → improved DFS
Lackner et al. [30]	70	II, III	CD68	Low CD68^+^ TAM number PT → decreased survival
Forssell et al. [31]	446	I, II, III, IV	CD68	High CD68^+^ TAM number PT → improved CSS
Li J et al. [32] Review and meta-analysis	6115	I, II, III, IV	CD68, iNOS, CD163	High CD68^+^ TAM number → improved OS iNOS^+^, CD163^+^ TAM number did not associate with survival
Edin et al. [33]	485	I, II, III, IV	NOS2^+^, CD163	High NOS2^+^ TAM number PT, high CD163^+^ number PT→ improved CSS
Koelzer et al. [34]	205	I, II, III, IV	CD68, iNOS, CD163	High CD68^+^ TAM number → improved OS High CD163^+^ TAM number → less lymph node metastases, absence of LVI, lower tumor grade
Väyrynen et al. [35]	931	I, II, III, IV	CD68, C86, IRF5, MRC1, MAF	High M1/M2 TAM ratio → improved CSS High M2 TAM number → shorter survival
Xue et al. [36]	209	I, II, III	CD163	High CD163^+^ TAM number → shorter DFS and OS
Inagaki et al. [37]	87	M0	CD68, CD163	High M2 TAM number, low M1 TAM number PT → more lymph node metastases
Yang et al. [38]	81	I, II, III	CD68, CD163	High CD163^+^/CD68^+^ ratio PT→ shorter RFS and OS

CRC, colorectal cancer; CSS, cancer-specific survival; DFS, disease-free survival; DSS, disease-specific survival; IT, intratumoral; LVI, lymphatic vessel invasion; OS, overall survival; PT, peritumoral; RFS, recurrence-free survival; TAM, tumor associated macrophage.

**Table 2:** Patient characteristics.

Characteristic	*n* = 498 (%)
Sex	
Female	237 (47.6)
Male	261 (52.4)
Stage of disease	
I	74 (14.9)
II	183 (36.7)
III	131 (26.3)
IV	110 (22.1)
Site of primary tumor	
Colon	269 (54%)
Rectum	229 (46%)
Sidedness of primary tumor	
Left sided	358 (71.9)
Right sided	140 (28.1)
Tumor differentiation grade	
Grade 1	16 (3.2)
Grade 2	321 (64.5)
Grade 3	138 (27.7)
Grade 4	23 (4.6)
Histological subtype	
Adenocarcinoma	426 (85.5)
Mucinous adenocarcinoma	72 (14.5)

**Table 3:** Intratumoral CD68^+^ and CLEVER-1^+^ macrophage and CLEVER-1^+^ lymphatic vessel score.

Macrophage and Lymphatic Vessel Score	*n* (%)
CD68^+^ macrophages	
0 (none, 0 macrophages/TMA spot)	34 (6.8)
1 (low, 1–29 macrophages/TMA spot)	61 (12.2)
2 (moderate, 30–99 macrophages/TMA spot)	73 (14.7)
3 (high, >100 macrophages/TMA spot)	305 (61.2)
Missing	25 (5.0)
CLEVER-1^+^ macrophages	
0 (none, 0 macrophages/TMA spot)	5 (1.0)
1 (low, 1–9 macrophages/TMA spot)	29 (5.8)
2 (moderate,10–30 macrophages/TMA spot)	64 (12.9)
3 (high, >30 macrophages/TMA spot)	352 (70.7)
Missing	48 (9.6)
CLEVER-1^+^ lymphatic vessels	
None (-)	17 (3.4)
A few (+)	86 (17.3)
Moderate (++)	132 (26.5)
Abundant (+++)	215 (43.2)
Missing	48 (9.6)

## Data Availability

The data presented in this study are available on reasonable request from the corresponding author.
